# Nonlinear threshold responses and spatial heterogeneity of soil organic carbon under contrasting pedoclimatic regimes

**DOI:** 10.3389/fpls.2025.1703663

**Published:** 2025-12-09

**Authors:** Jin Cui, Yuzhi Xu, Mengqi Wang, Aiju Liu, Lin Sun, Xinyu Feng, Qingrun Yang, Shang Wang, Hongqiang Liu, Yujuan Lv, Kai Liu

**Affiliations:** 1 School of Resources and Environmental Engineering, Shandong University of Technology, Zibo, China; 2 Zibo Key Laboratory of Agricultural Soil and Water Environmental Pollution Control, Zibo, Shandong, China; 3 Department of Soil Ecology, Helmholtz-Centre for Environmental Research-UFZ, Halle (Saale), Germany; 4 State Key Laboratory of Efficient Utilization of Arable Land in China (the Institute of Agricultural Resources and Regional Planning), Chinese Academy of Agricultural Sciences, Beijing, China; 5 Central South Academy of Inventory and Planning of National Forestry and Grassland Administration, Changsha, Hunan, China; 6 Nanjing Institute of Environmental Sciences, Ministry of Ecology and Environment, Nanjing, China

**Keywords:** soil organic carbon, geostatistics, spatial heterogeneity, machine learning models, cropland management

## Abstract

Soil organic carbon (SOC) exhibits distinct spatial heterogeneity across different pedoclimatic regions, yet the underlying regulatory mechanisms and their threshold responses remain poorly understood. In this study, the spatial patterns and underlying region-specific regulatory factors controlling SOC dynamics were investigated across a pedoclimatic gradient represented by the Jiaodong Peninsula (maritime monsoon climate) and Southwest Shandong (continental climate) in Shandong Province, China. Geostatistical analysis coupled with sequential Gaussian simulations provided probabilistic assessment of SOC spatial patterns, while machine learning algorithms (Linear Regression, Random Forest, XGBoost and Support Vector Machine) integrated with SHAP analysis enabled quantification of nonlinear threshold responses and identification of dominant factors governing SOC dynamics. The results showed that SOC in Jiaodong exhibited a west-high-east-low gradient characterized by local-scale structure, whereas Southwest Shandong showed higher SOC contents dominated by macro-scale gradients. The Random Forest model identified distinct regulatory mechanisms in Jiaodong, where NO_3_^−^-N and extractable Fe exhibited a dual-threshold domain (NO_3_^−^-N = 10.0 mg·kg^−1^, Fe = 12.0 mg·kg^−1^), with the marginal effect of Fe on SOC shifting from negative to positive when NO_3_^−^-N exceeded its threshold concentration. In Southwest Shandong, total nitrogen (TN) was revealed as the dominant predictor, with a critical threshold at 3.25 g·kg^−1^ above which SOC increased by 2.0 g·kg^−1^, while NO_3_^−^-N showed negative effects above 27 mg·kg^−1^. This study demonstrates that the combination of interpretable machine learning and geostatistical approaches can effectively elucidate region-specific threshold mechanisms and nonlinear controls governing SOC dynamics. This approach is critical for developing spatially-explicit soil carbon management strategies under varying pedoclimatic conditions.

## 1 Introduction

Soil organic carbon (SOC) constitutes the largest terrestrial carbon pool, storing approximately 1,500 Pg of carbon in the top meter of soil and playing a key role in regulating climate-carbon feedbacks through its effect on atmospheric CO_2_ concentrations (Lal, 2004; Schimel et al., 2015; Gleixner, 2013). In addition, SOC is fundamental to the provision of essential ecosystem services, such as nutrient cycling, water retention, soil aggregate stability, and biodiversity conservation (Lehmann and Kleber, 2015; Lal, 2018; Wiesmeier et al., 2019). However, SOC stocks are neither spatially uniform nor temporally stable. Substantial heterogeneity in SOC distribution and persistence across multiple scales and pedoclimatic regions represent a structural uncertainty-up to ±30% in terrestrial carbon flux estimates used in CMIP6 and Earth system models (Doetterl et al., 2015; Wang et al., 2021; Sanderson et al., 2024). This uncertainty is further increased by the non-linear and context-dependent responses of SOC to environmental drivers such as nitrate nitrogen (NO_3_^-^-N), extractable iron availability, and the interacting effects of total nitrogen (TN) with cation-exchange capacity (CEC) (Song et al., 2023). For example, the continental croplands on the North China Plain have undergone decades of intensive N fertilization, resulting in persistent soil and vadose-zone nitrate accumulation and leaching, whereas abundant CaCO_3_ and elevated cation exchange capacity (CEC) promote the stabilization of mineral-associated organic carbon (Ju et al., 2006; Zhou et al., 2016; Rowley et al., 2018a). In contrast, in coastal monsoonal landscapes of eastern China, periodic hydrological redox fluctuations drive active transformations among Fe phases, thereby influencing the dynamics of Fe-organic associations (Wang et al., 2019; Zhao et al., 2022, 2023). Reducing these uncertainties is not only essential for improving model reliability but also foundational to achieving international climate targets outlined in the IPCC Sixth Assessment Report and initiatives like the “4 per 1000” strategy for soil carbon sequestration (Minasny et al., 2017; Paustian et al., 2016).

To understand and reduce these uncertainties requires disentangling how SOC dynamics respond to contrasting pedoclimatic conditions and the mechanisms underlying such variability. Regionally, the SOC stability is governed by nonlinear interactions among climatic gradients, soil physicochemical attributes, and nutrient availability, resulting in divergent stabilization mechanisms across landscapes (Doetterl et al., 2015; Wang et al., 2021; Xu and Tsang, 2022). In humid coastal regions subject to maritime-monsoon climates, frequent redox oscillations driven by fluctuating moisture regimes and high organic carbon inputs accelerate SOC turnover through microbial mineralization and the formation of organo-Fe complexes (Keiluweit et al., 2015; Yu et al., 2021). These regions are characterized by elevated microbial biomass, acidic pH, and high dissolved organic carbon concentrations, all of which enhance SOC solubility and mobility while simultaneously reducing long-term mineral protection due to reduced sorption affinity and organo-mineral interactions (Rumpel and Kögel-Knabner, 2011; Keiluweit et al., 2017; Kramer and Chadwick, 2018). In contrast, in semi-arid continental regions with limited precipitation and alkaline soils, SOC stabilization is dominated by persistent mineral associations, including cation bridging, calcium-carbonate interactions, and selective sorption onto clay minerals (Kleber et al., 2007; Liang et al., 2019). Such pedoclimatic contrasts give rise to distinct biogeochemical thresholds and feedback mechanisms that are poorly represented in current Earth-system models, which typically oversimplify SOC responses to nutrient enrichment and climate variability (Guenet et al., 2018; Wang et al., 2021). Therefore, region-specific frameworks that explicitly account for mineral protection and nutrient-coupled destabilization processes are vital for improving model predictability across diverse soil-climate regimes.

Despite substantial advances in identifying dominant controls of SOC dynamics, such as mineral protection, nitrogen availability, and soil physicochemical properties, the non-linear thresholds that regulate SOC stability under varying nutrient availability across climatic regimes remain poorly understood. For instance, nitrate nitrogen (NO_3_^-^-N) and extractable iron (Fe) have been shown to exhibit highly context-specific effects, whereby they enhance SOC stabilization through organo-mineral associations in humid, redox-fluctuating soils, yet promote carbon loss in drier systems via altered microbial functioning and reduced mineral sorption (Keiluweit et al., 2015; Ruiz et al., 2024; Lambert et al., 2024; Díaz-Martínez et al., 2024). Moreover, interactions among multiple controlling factors, such as NO_3_^-^-N, Fe oxides, and cation exchange capacity, typically lead to nonlinear reinforcing or offsetting effects that are poorly quantified across pedoclimatic gradients with contrasting mineralogy, nutrient availability, and microbial community composition (Liang et al., 2019; Wang et al., 2021; Chen et al., 2025). Conventional linear models have shown limited ability to capture these complex, nonlinear dynamics of SOC, especially in the presence of interacting environmental drivers (Doetterl et al., 2015; Guenet et al., 2018; Wang et al., 2021). Therefore, advanced analytical frameworks, such as sequential Gaussian simulations combined with probabilistic spatial analysis, provide robust approaches for quantifying spatial uncertainty and characterizing region-specific SOC variability (Yu et al., 2021; Xu and Tsang, 2022; Wang et al., 2021). Meanwhile, machine learning models, such as Random Forest, XGBoost and Support Vector Machines, combined with SHAP (SHapley Additive exPlanations) analysis, can effectively identify nonlinear threshold responses and multi-factor interactions, thereby enhancing both the mechanistic interpretability and predictive capacity of SOC models across contrasting pedoclimatic conditions (Liang et al., 2019; Huang et al., 2025; Kakhani et al., 2025).

Building on these methodological advances, this study examines the distinct climatic and edaphic contrasts between the Jiaodong Peninsula (maritime monsoon climate) and Southwest Shandong (continental climate) as a natural model system to investigate SOC spatial heterogeneity and its underlying regulatory mechanisms. Using a probability-based geostatistical framework combined with machine learning approaches, we first quantified SOC spatial uncertainty through sequential Gaussian simulation and probabilistic spatial analysis, and subsequently integrated Random Forest with SHAP and partial-dependence analysis to reveal interpretable nonlinear response patterns. Accordingly, we tested two hypotheses: (1) The spatial distribution of SOC differs fundamentally between the Jiaodong Peninsula (maritime monsoon) and Southwest Shandong (continental); (2) The dominant factors regulating SOC stability exhibit distinct region-specific controls in these contrasting environments. By integrating advanced spatial analysis with interpretable machine learning approaches, this study aims to elucidate the mechanisms underlying regional SOC dynamics, thereby providing a framework for region-specific sustainable soil management strategies under accelerating climate change.

## 2 Materials and methods

### 2.1 Site description and soils collection

This study was conducted in two contrasting agricultural regions of Shandong Province, China: The Jiaodong Peninsula (Yantai, Weihai, and Qingdao) and Southwest Shandong (Jining and Zaozhuang). The Jiaodong Peninsula is dominated by low mountains and hilly landforms under a warm-temperate monsoon climate (mean annual temperature (MAT) 12.5 °C; mean annual precipitation (MAP) 630–1114 mm). Southwest Shandong comprises a transitional zone from the Huang-Huai-Hai Plain to the central-southern highlands with a continental warm-temperate monsoon climate (MAP 616–880 mm). A total of 48 and 41 macro-sites were established in the Jiaodong Peninsula and Southwest Shandong, respectively, to encompass the principal pedoclimatic, geomorphic, and edaphic gradients characteristic of each region. The sampling framework systematically integrated climatic conditions, topography, and soil classification to capture the dominant combinations of temperature, precipitation, and parent material that define the regional pedoenvironments. Within each region, sites were distributed across the full range of mean annual precipitation and elevation gradients, including the main FAO/WRB soil groups such as Cambisols, Luvisols, Fluvisols, and Anthrosols. All selected croplands exhibited comparable management histories, characterized by long-term (>10 years) winter wheat-summer maize rotation under similar fertilization and irrigation regimes, and sites affected by recent disturbance, construction, or land-use conversion were excluded. This stratified design ensured that the collected samples captured the principal environmental gradients controlling soil organic carbon variability across the contrasting pedoclimatic regimes.

Soil sampling was conducted in May 2021, coinciding with the grain-filling stage of winter wheat, when fields were typically irrigated but not fertilized. At each site, three 200 m × 200 m grids were established, from which five topsoil cores (0–20 cm) were collected using a five-point pattern, yielding 144 samples in the Jiaodong Peninsula and 123 in Southwest Shandong. All samples were air-dried at room temperature (25 °C) for 7 days, gently disaggregated, cleared of visible plant residues and gravel, passed through a 2-mm nylon sieve, and stored in sealed polyethylene bags prior to analysis.

### 2.2 Soil and environmental variables

Soil properties were determined following national standard protocols (Lu, 1999). Soil organic carbon (SOC) was quantified by external-heating K_2_Cr_2_O_7_ oxidation. Soil pH was measured potentiometrically in a 1:2.5 (w/v) soil-water suspension (Mettler-Toledo FE28, Switzerland). Mineral nitrogen was determined after 2 mol L^-^¹ KCl extraction: nitrate (NO_3_^-^-N) by UV spectrophotometry and ammonium (NH_4_^+^-N) by indophenol blue colorimetry. Total nitrogen (TN) was measured by the Kjeldahl method, and total phosphorus (TP) by H_2_SO_4_-HClO_4_ digestion followed by molybdenum-antimony colorimetry. Cation exchange capacity (CEC) was determined by the 1 mol L^-^¹ NH_4_OAc (pH 7.0) exchange method. Exchangeable Ca²^+^ and Mg²^+^ were quantified by atomic absorption spectrophotometry (AAS) after NH_4_OAc extraction. DTPA-extractable Fe was determined using AAS following extraction with a DTPA-CaCl2-TEA solution. Topographic variables, such as elevation (ELE), slope (SLOPE), and aspect (ASP), were derived from a 30-m resolution Digital Elevation Model (DEM) sourced from the Geospatial Data Cloud. Climatic variables (MAT and MAP) were calculated as averages from 2020–2022 meteorological station data obtained from the China Meteorological Data Service Centre. All raster covariates were co-registered to the DEM grid before their values were extracted to the sampling locations.

### 2.3 Geostatistical modeling and probabilistic mapping of SOC

The spatial structure of SOC was assessed using semivariograms, and prediction surfaces were generated via ordinary kriging (OK). Prior to variogram modeling, data were checked for normality and transformed (log or Box-Cox) where necessary. Spherical, exponential, and Gaussian models were fitted, with the final model choice and its parameters (nugget, sill, range) selected based on leave-one-out cross-validation to minimize RMSE. Detailed semivariogram parameters including model type, nugget, sill, range and cross-validation statistics were provided in **Supplementary Table S1**. To quantify spatial uncertainty, the sequential Gaussian simulation (SGS) was implemented. Data were transformed to normal scores, simulated on the prediction grid within a moving neighborhood defined by the fitted variogram, and then back-transformed. An ensemble of 1,000 conditional realizations yielded pixel-wise means and standard deviations, enabling robust assessment of spatial uncertainty.

To probabilistically assess the magnitude and spatial pattern of SOC differences between the two regions, we calculated pixel-wise differences between corresponding realizations from the two SGS ensembles (defining ΔSOC = SOC_Southwest - SOC_Jiaodong). This process yielded an ensemble of 1,000 ΔSOC maps. From this distribution of 1,000 ΔSOC values at each pixel, we then calculated the probability that the difference would fall within predefined intervals (e.g., >20, 10 to 20,…, < -6 g·kg^-^¹). This approach allowed for the quantification of robust probability maps of regional difference.

### 2.4 Machine learning modeling and interpretation

To identify dominant controls on SOC and reveal nonlinear responses, four machine learning models were trained: Linear Regression (LR), Random Forest (RF), Support Vector Machine with a radial basis function kernel (SVM-RBF), and eXtreme Gradient Boosting (XGBoost). The predictor set comprised the measured soil properties and the topographic/climatic covariates. Continuous predictors were z-standardized for LR and SVM. The dataset was split into independent training (70%) and testing (30%) sets, stratified by region. Hyperparameters for RF (e.g., n_estimators, mtry), SVM (e.g., C, gamma), and XGBoost (e.g., n_estimators, learning_rate, max_depth) were optimized using a grid search nested within 10-fold cross-validation on the training set.

To identify numerical thresholds in the relationships between soil and environmental variables and SOC, a prespecified two-stage analytical procedure was applied. First, partial-dependence functions and SHAP dependence plots from the best-performing models were analyzed to define each predictor-response form and to locate candidate breakpoints, operationally identified as local extrema or curvature changes in the PDPs, or as directional shifts in SHAP contributions. Second, candidate values were tested by fitting segmented regressions to cross-validated model predictions with locally weighted smoothing (LOESS) to stabilize fits. The optimal threshold was the breakpoint that minimized the residual sum of squares (RSS) and improved explanatory power relative to the unsegmented fit. This procedure produced statistically robust, data-constrained inflection points in the identification of nonlinear transitions in SOC dynamics.

Model interpretation centered on the best-performing models. We computed permutation-based feature importance and utilized SHAP (SHapley Additive exPlanations) to decompose feature contributions. SHAP values were calculated using the TreeExplainer algorithm for the machine learning models, with interaction effects computed for all pairwise combinations of the top five predictors. SHAP values represent the contribution of each feature to the deviation from the expected model output. Partial Dependence Plots (PDPs) visualized the marginal effects of key predictors, while SHAP interaction values were used to diagnose bivariate thresholds and synergistic or antagonistic effects (e.g., NO_3_^-^-N × Fe; TN × CEC).

### 2.5 Model evaluation and reproducibility

The predictive performance of the machine learning models was evaluated on the held-out test sets using the adjusted coefficient of determination (adj R²) and root mean square error (RMSE). The accuracy of kriging models was assessed via leave-one-out cross-validation metrics. All geostatistical analyses were conducted in ArcGIS 10.8, while machine learning, statistical analyses, and graphics were performed in Python 3.9 (using scikit-learn, xgboost, shap) and R 4.2.0 (using gstat, ggplot2).

## 3 Results

### 3.1 Spatial heterogeneity of SOC in the Jiaodong Peninsula and Southwest Shandong

**Figure 1:**
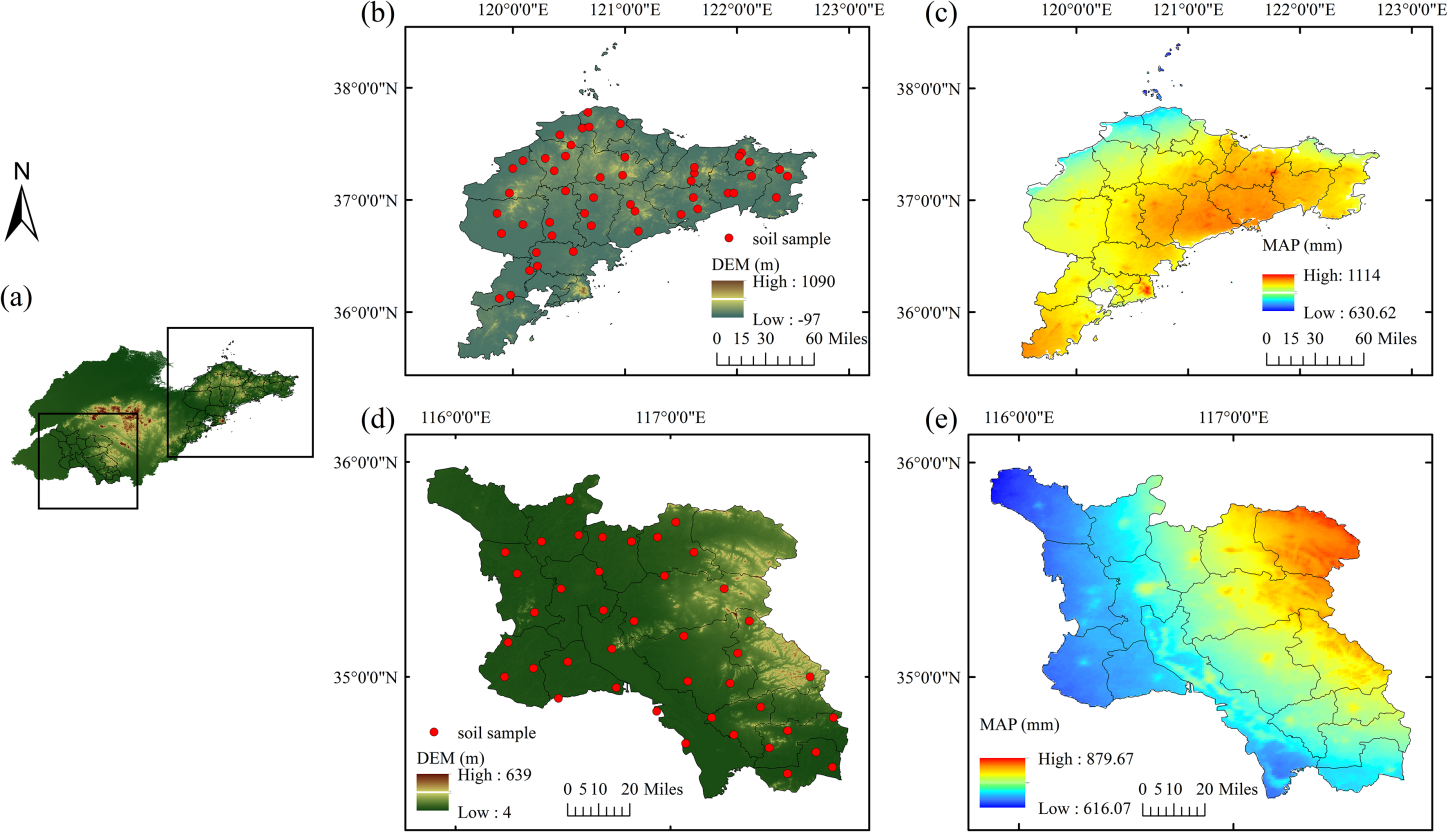
Distribution of sampling sites in the study regions: **(a)** digital elevation model (DEM) of Shandong Province; **(b)** sampling locations in the Jiaodong Peninsula, Shandong Province; **(c)** mean annual precipitation in the Jiaodong Peninsula, Shandong Province; **(d)** DEM and sampling locations in Southwest Shandong Province; **(e)** mean annual precipitation in Southwest Shandong Province.

**Figure 2:**
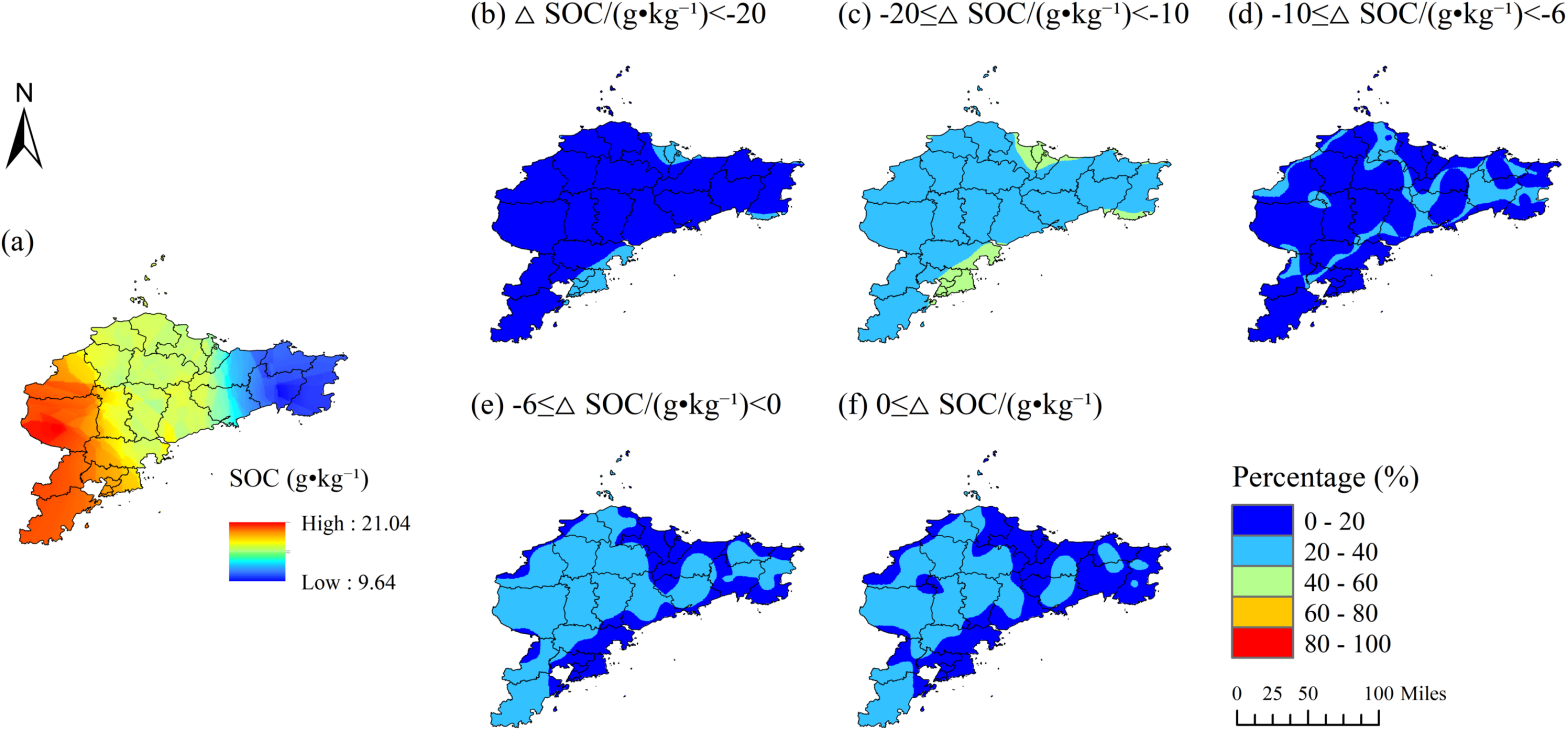
Probability distribution of relative soil organic carbon (SOC) differences between the Jiaodong Peninsula and Southwest Shandong, Shandong Province: **(a)** distribution of soil organic carbon (SOC; g·kg^-1^) in the Jiaodong Peninsula; **(b)** probability of ≥SOC < -20 g·kg^-^¹; **(c)** probability of -20 ≤ ≥SOC < -10 g·kg^-^¹; **(d)** probability of -10 ≤ ≥SOC < -6 g·kg^-^¹; **(e)** probability of -6 ≤ ≥SOC < 0 g·kg^-^¹; **(f)** overall probability of ≥SOC ≥ 0.

**Figure 3:**
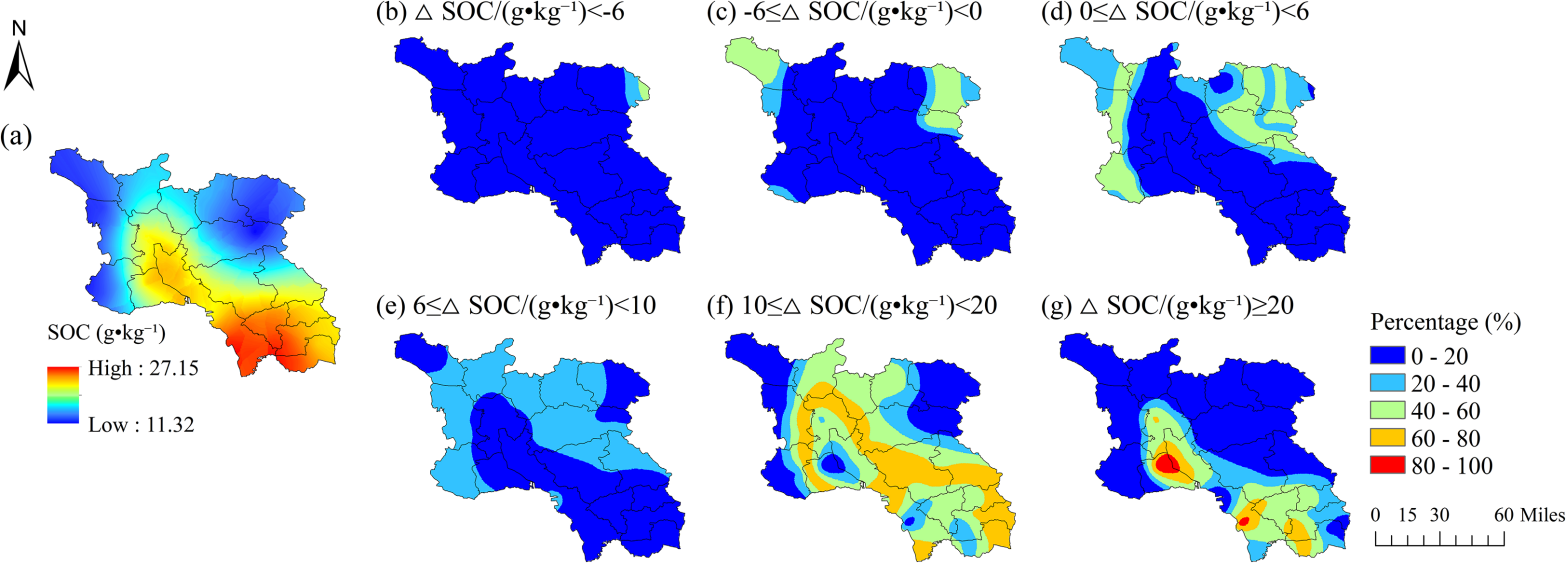
Probability distribution of relative soil organic carbon (SOC) differences between the Southwest Shandong and the Jiaodong Peninsula, Shandong Province: **(a)** distribution of soil organic carbon (SOC; g·kg^-1^) in the Southwest Shandong; **(b)** probability of ≥SOC < -6 g·kg^-^¹; **(c)** probability of -6 ≤ ≥SOC < 0 g·kg^-^¹; **(d)** probability of 0 ≤ ≥SOC < 6 g·kg^-^¹; **(e)** probability of 6 ≤ ≥SOC < 10 g·kg^-^¹; **(f)** probability of 10 ≤ ≥SOC < 20 g·kg^-^¹; **(g)** probability of ≥SOC ≥ 20 g·kg^-^¹.

The distribution of SOC in Jiaodong displays a west-high-east-low gradient (9.64-21.04 g·kg^-^¹; **Figures 1**; **2a**), spatially coincident with elevated Fe content and low NO_3_^-^-N levels (<10 mg·kg^-^¹; **Supplementary Figure S1**). Probability-based spatial analysis reveals that over 80% of the Jiaodong Peninsula has a 40-60% probability of SOC being 10–20 g·kg^-^¹ lower than those in Southwest Shandong (**Figure 2c**), with <20% probability of equal or higher values (**Figure 2f**). In contrast, Southwest Shandong exhibits higher SOC (11.32-27.15 g·kg^-^¹), peaking in the southeastern hilly zones, where TN (>4 g·kg^-^¹) and CEC (>12 cmol·kg^-^¹) jointly enhance SOC accumulation (**Figures 3a–c**). In addition, 60-80% of the area shows >60% probability of SOC contents exceeding those in Jiaodong by 10–20 g·kg^-^¹ (**Figure 3f**), while localized hotspots (≤10% of the area) have >80% probability of ≥SOC ≥20 g·kg^-^¹ (**Figure 3g**). Semivariogram analysis further reveals that SOC variability in Jiaodong is regulated by fine-scale heterogeneity in soil properties or nutrients (range = 42.6 km, nugget = 39.14%), whereas in Southwest Shandong, macro-scale gradients dominate SOC patterns (range = 68 km, nugget = 1.12%) (**Supplementary Table S1**).

### 3.2 Spatial uncertainty and stability of SOC across contrasting pedoclimatic regions

**Figure 4:**
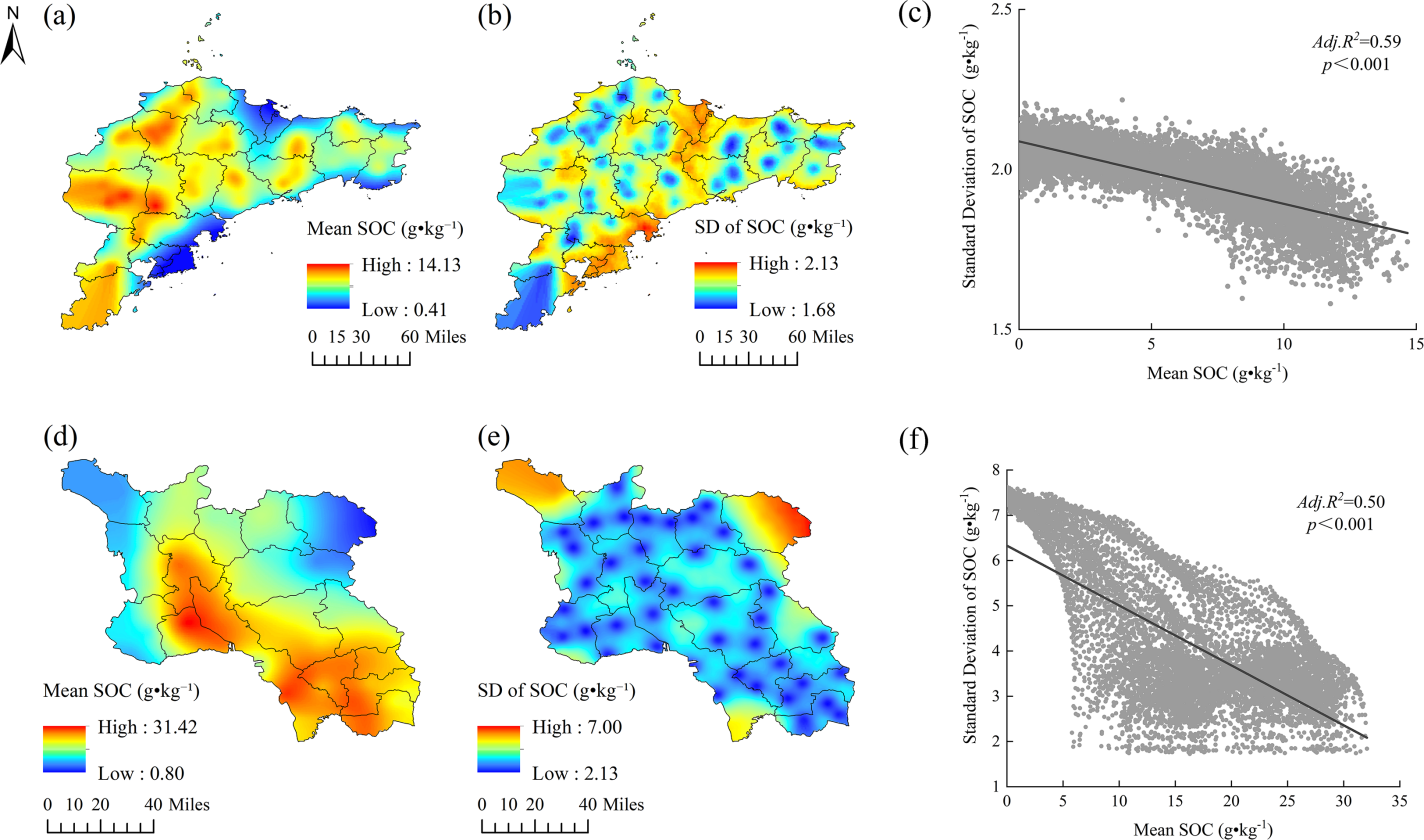
Spatial distribution and statistical relationships of soil organic carbon (SOC) in Shandong Province: **(a)** mean SOC in the Jiaodong Peninsula derived from ordinary kriging interpolation; **(b)** standard deviation of SOC in the Jiaodong Peninsula; **(c)** mean SOC in Southwest Shandong derived from ordinary kriging interpolation; **(d)** standard deviation of SOC in Southwest Shandong; **(e)** linear regression between mean and standard deviation of SOC in the Jiaodong Peninsula; **(f)** linear regression between mean and standard deviation of SOC in Southwest Shandong.

Sequential Gaussian simulations provide deeper insights into spatial uncertainty and stability of SOC across regions. The Jiaodong Peninsula displays a distinct west-high/east-low pattern in the mean values of SOC, with standard deviations highest in low-SOC coastal zones (**Figures 4a,b**). Linear regression analyses revealed a strong inverse relationship between the mean values of SOC and standard deviation (Adj. R² = 0.59, *p* < 0.001; **Figure 4c**), indicating that SOC-rich areas (e.g., western hills) exhibit greater stability of SOC stocks, while coastal zones are more heterogeneous. In contrast, the spatial variability of SOC was higher in Southwest Shandong, where the mean values are highest in the southeast (up to 31.42 g·kg^-^¹) and the standard deviations (up to 7.00 g·kg^-^¹) are greatest in the nutrient-deficient northwestern plains (**Figures 4d,e**). The significant negative correlation between SOC mean values and their corresponding standard deviations (Adj. R² = 0.50, p < 0.001; **Figure 4f**) suggests that areas with higher SOC contents tend to exhibit lower spatial variability. Notably, SOC uncertainty in Southwest Shandong exhibits a nonlinear threshold response to total nitrogen (TN), with a sharp discontinuity around 3.25 g·kg^-^¹ TN (**Figures 4d–f**). Below this critical threshold (TN < 3 g·kg^-^¹), SOC prediction uncertainty increased substantially (SD > 5 g·kg^-^¹), reflecting enhanced micro-scale spatial heterogeneity of N-limited systems. In contrast, high nitrogen availability (TN > 4 g·kg^-^¹) reduced the spatial variability of SOC (SD < 2.5 g·kg^-^¹), highlighting the dominant control of the nitrogen availability on the spatial predictability of SOC.

### 3.3 Nonlinear threshold responses of SOC by nitrate nitrogen and extractable Fe in Jiaodong Peninsula Shandong

**Figure 5:**
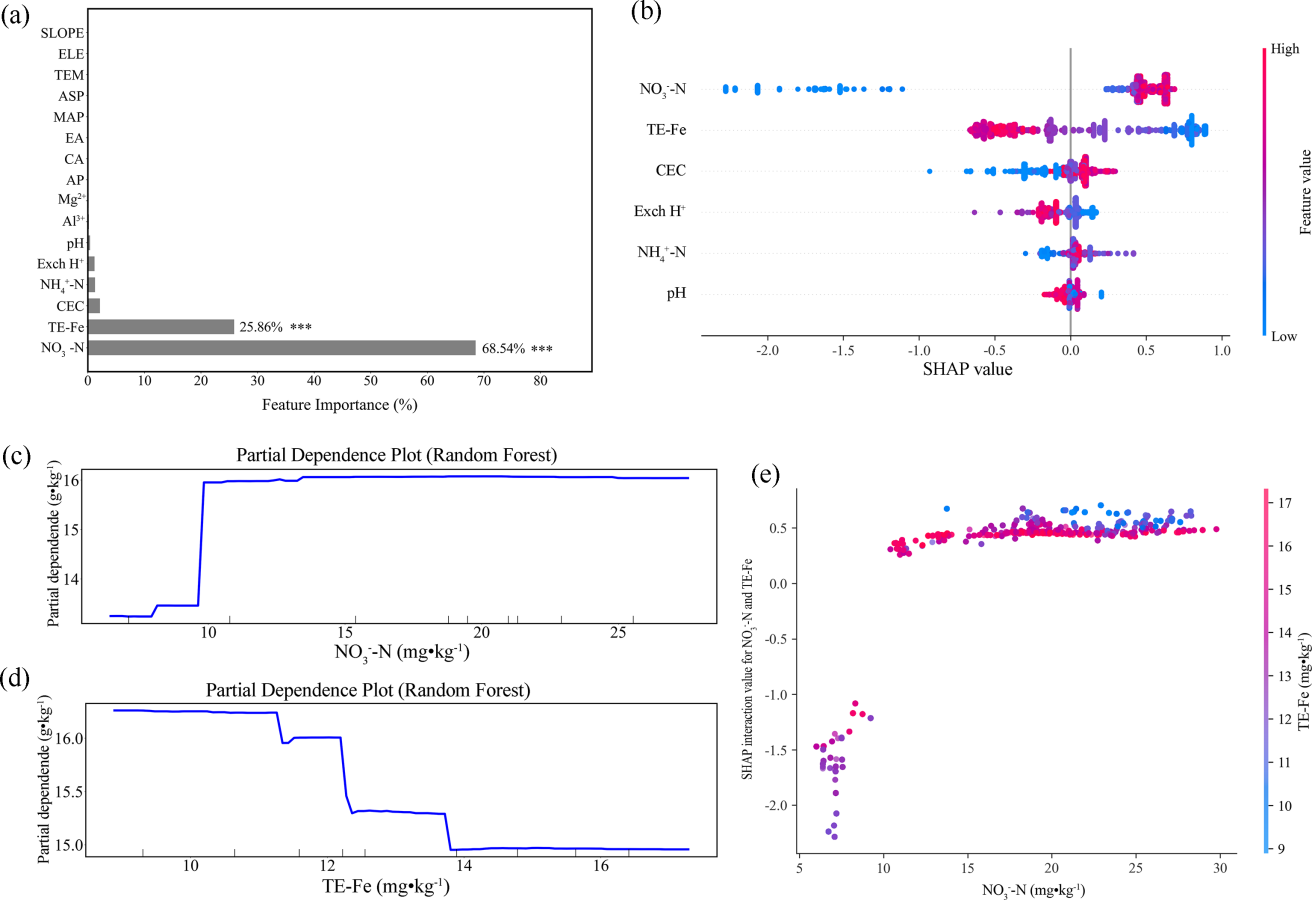
Random forest analysis of the dominant factors controlling soil organic matter in the Jiaodong Peninsula: **(a)** Variable importance ranking of soil physicochemical properties, indicating nitrate nitrogen (NO_3_^-^-N) and extractable iron (Fe) as the dominant predictors (***p < 0.001); **(b)** SHAP summary plot of the random forest model, showing the relative contributions of NO_3_^-^-N, extractable Fe, cation exchange capacity (CEC), exchangeable hydrogen (Exch H^+^), ammonium nitrogen (NH_4_^+^-N) and hydrogen ion concentration (pH); **(c)** Partial dependence plot (PDP) illustrating the marginal effect of NO_3_^-^-N on soil organic matter; **(d)** Partial dependence plot (PDP) illustrating the marginal effect of extractable Fe on soil organic matter; **(e)** SHAP interaction values showing the interactive effect between NO_3_^-^-N and extractable Fe.

In the Jiaodong Peninsula, feature importance analysis identified nitrate nitrogen (NO_3_^-^-N) and extractable iron as the dominant predictors of SOC dynamics, explaining 68.3% and 24.1% of the model variance, respectively (**Figure 5a**). This is further supported by SHAP analysis, which revealed a positive association between elevated NO_3_^-^-N (>10 mg·kg^-^¹) and SOC accumulation (ΔSHAP = +0.82) (**Figure 5b**). This threshold-dependent pattern was further elucidated by partial dependence analysis, which indicated a distinct nonlinear response, where SOC remained largely invariant (13.5 ± 0.4 g·kg^-^¹) below 10 mg·kg^-^¹ NO_3_^-^-N, and increased sharply at this threshold, and subsequently reached a steady state at 16.0 g·kg^-^¹, suggesting constraints on NO_3_^-^-N-mediated SOC stabilization (**Figure 5c**). In contrast, extractable iron exhibited a concentration-dependent biphasic effect on SOC stability. At concentrations below 11.5 mg·kg^-^¹, iron enhanced SOC retention (SHAP: +0.48 to +0.80), whereas values exceeding this threshold induced negative effects, with SHAP values declining to -0.8 ± 0.15 (**Figures 5b, d**). Notably, SHAP interaction analysis revealed a synergistic relationship between NO_3_^-^-N and extractable iron (**Figure 5e**). When extractable Fe exceeded 12.0 mg·kg^-^¹, the marginal effect of NO_3_^-^-N on SOC shifted from strongly inhibitory (SHAP: -2.2 to -1.0) to neutral or slightly positive (SHAP: 0 to +0.5) upon crossing its 10.0 mg·kg^-^¹ threshold. This “dual-threshold domain” (NO_3_^-^-N = 10.0 mg·kg^-^¹ × Fe = 12.0 mg·kg^-^¹) indicates a critical biogeochemical transition zone, where NO_3_^-^-N availability appears to mediate the directional switch in iron’s regulatory role in SOC stability.

### 3.4 Nonlinear threshold responses of SOC by nitrogen, phosphorus, and cation exchange capacity in Southwestern Shandong

**Figure 6:**
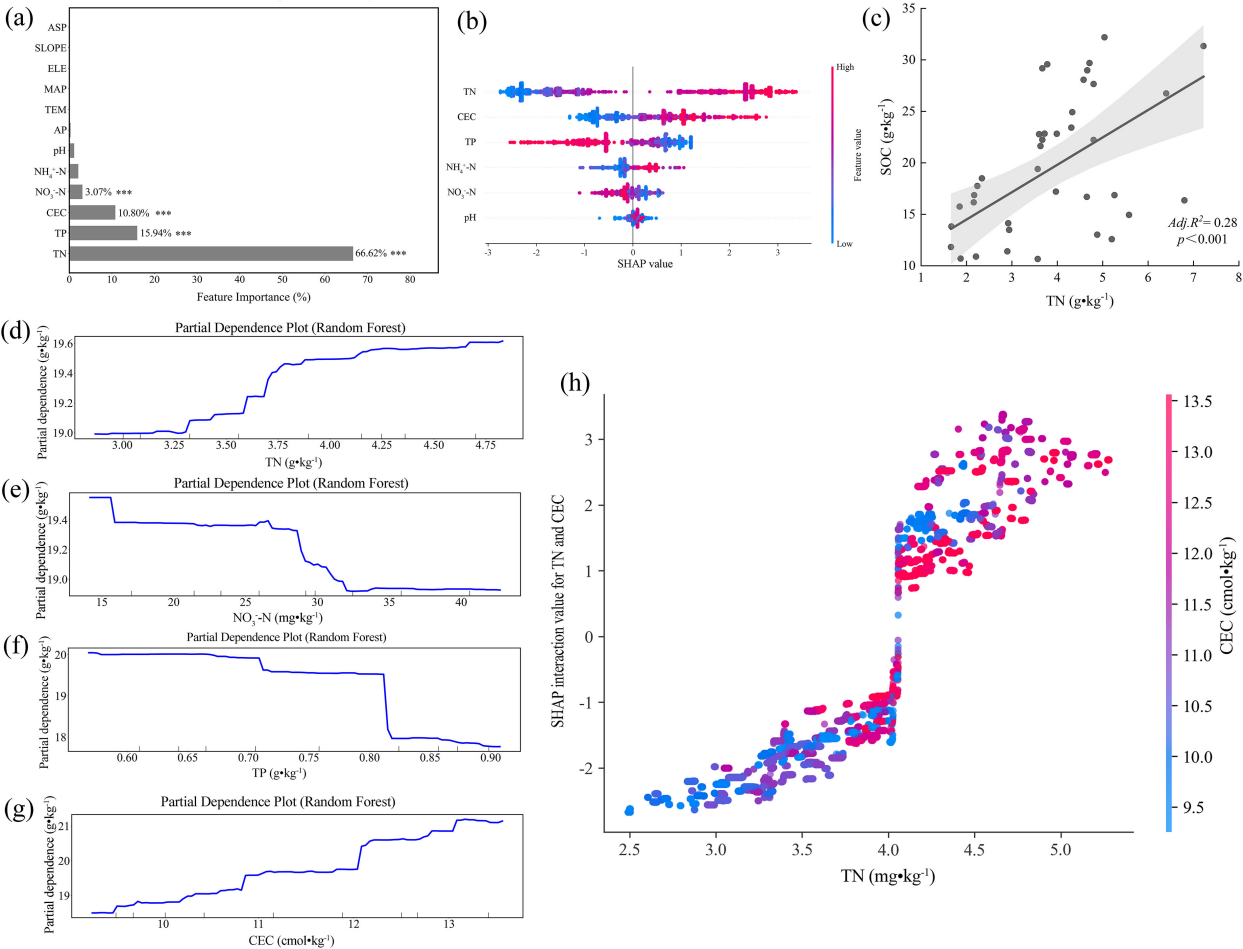
Random forest analysis of the dominant factors controlling soil organic matter in Southwest Shandong: **(a)** The variable importance ranking of soil physicochemical properties, indicating total nitrogen (TN), total phosphorus (TP), cation exchange capacity (CEC), and nitrate nitrogen (NO_3_^-^-N) as the dominant predictors (***p < 0.001); **(b)** The SHAP summary plot of the random forest model, showing the relative contributions of TN, CEC, TP, ammonium nitrogen (NH_4_^+^-N), NO_3_^-^-N, and hydrogen ion concentration (pH); **(c)** The linear regression between soil organic carbon (SOC) and TN; **(d)** The partial dependence plot (PDP) illustrating the marginal effect of TN on SOC; **(e)** The PDP illustrating the marginal effect of NO_3_^-^-N on SOC; **(f)** The PDP illustrating the marginal effect of TP on SOC; **(g)** The PDP illustrating the marginal effect of CEC on SOC; **(h)** The SHAP interaction values showing the interactive effect between TN and CEC.

In the continental agroecosystems of Southwestern Shandong, total nitrogen (TN) was determined as the dominant predictor of SOC dynamics (67.12% of the model variance; **Figures 6a,b**). Linear regression exhibited a significant positive relationship (adjusted R² = 0.28, β = 2.89, p < 0.001; **Figure 6c**), while partial dependence analysis revealed a critical threshold at 3.25 g·kg^-^¹ TN, above which SOC increased sharply from 17.5 to 19.5 g·kg^-^¹ (**Figure 6d**). Given that the regional median TN (3.67 g·kg^-^¹) marginally exceeds the identified threshold, almost half of the soils remain functionally nitrogen-limited with respect to SOC accumulation potential. In contrast, nitrate nitrogen (NO_3_^-^-N) exhibited a pronounced negative threshold effect, whereby SOC declined by 3.1% (*P* < 0.01) when NO_3_^-^-N exceeded 27 mg·kg^-^¹ (**Figure 6e**). This pattern was further supported by SHAP values, which revealed strongly negative marginal contributions (-1.2) at concentrations above 40 mg·kg^-^¹ (**Figure 6b**). Meanwhile, total phosphorus (TP) exhibited a distinctive dual-threshold suppression pattern, where SOC decreased by 2.0–4.0 g·kg^-^¹ across sequential thresholds (0.71 and 0.81 g·kg^-^¹; **Figure 6f**). Given that the regional median NO_3_^-^-N (14.94 mg kg^-^¹) and TP (0.71 g kg^-^¹) were close to their respective critical thresholds for SOC stability, a dual high-risk domain characterized by co-elevated NO_3_^-^-N and TP levels was identified. In addition, cation exchange capacity (CEC) also displayed a positive effect on SOC, characterized by two distinct response thresholds (**Figure 6g**). An initial SOC increase (+6.7%, P < 0.001) occurred at CEC = 10.8 cmol·kg^-^¹, and followed by a secondary rise at 12.1 cmol kg^-^¹ that further elevated SOC by 2.2 g·kg^-^¹. SHAP interaction analysis demonstrated that TN and CEC co-regulate SOC with coupled constraints under nitrogen limitation and insufficient cation retention (**Figure 6h**). When total nitrogen (TN) concentrations fell below 4.0 g·kg^-^¹, the interactive effects of TN and cation exchange capacity (CEC) on SOC were predominantly negative (SHAP: -2.5 to 0). Once the critical TN threshold was exceeded, the interaction shifted sharply toward a positive direction (SHAP: +1.5 to +3.0), with elevated CEC levels substantially enhancing the contribution of TN to SOC stabilization.

### 3.5 Machine learning models reveal differential SOC prediction performance across contrasting pedoclimatic regions

**Figure 7:**
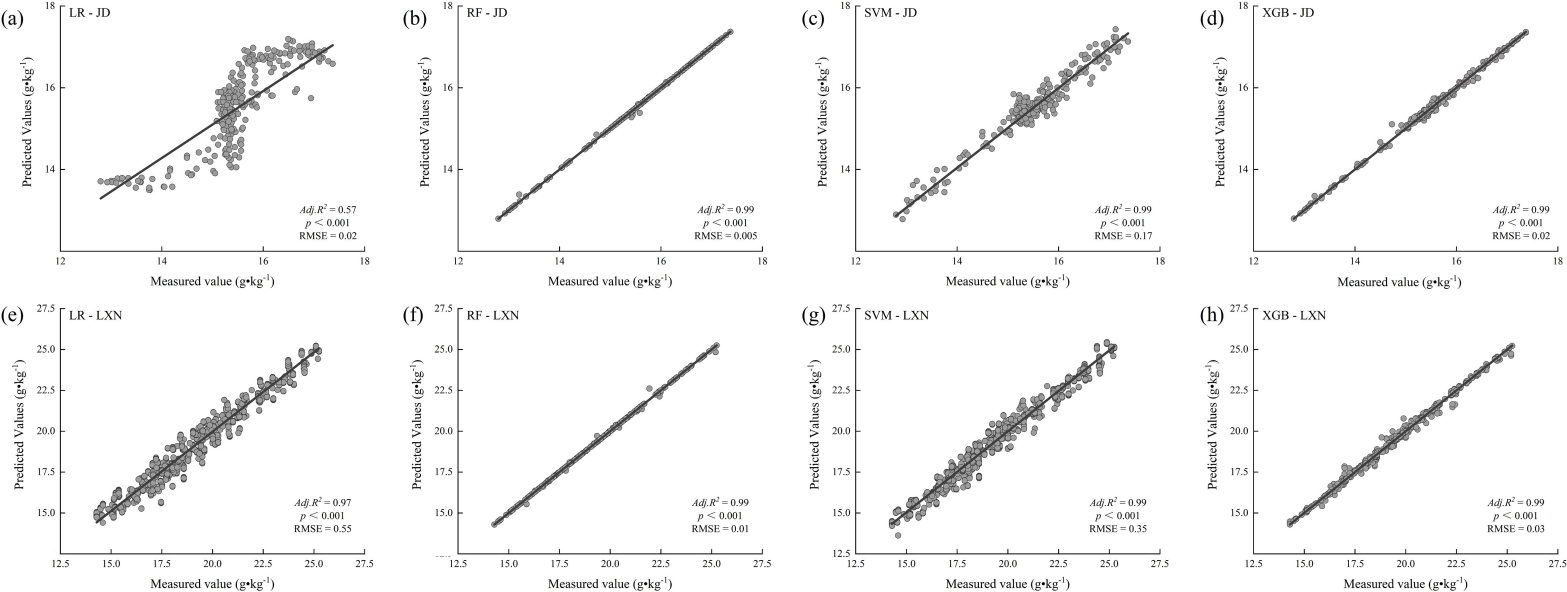
Model-based predictions of soil organic carbon (SOC) using different machine learning algorithms in the Jiaodong Peninsula (JD) and Southwest Shandong (LXN): **(a)** Linear regression (LR) for the Jiaodong Peninsula; **(b)** random forest (RF) for the Jiaodong Peninsula; **(c)** support vector machine (SVM) for the Jiaodong Peninsula; **(d)** XGBoost (XGB) for the Jiaodong Peninsula; **(e)** linear regression (LR) for Southwest Shandong; **(f)** random forest (RF) for Southwest Shandong; **(g)** support vector machine (SVM) for Southwest Shandong; **(h)** XGBoost (XGB) for Southwest Shandong.

Machine learning model evaluation across the Jiaodong Peninsula (maritime climate) and Southwestern Shandong (continental climate) identified Random Forest (RF) as the most effective predictor of soil organic carbon (SOC) dynamics, achieving high accuracy in both regions (Jiaodong: adjusted R² = 0.99, RMSE = 0.005; Southwest: adjusted R² = 0.99, RMSE = 0.01; **Figure 7**). This performance underscores RF’s strength in modeling complex, non-linear relationships inherent in heterogeneous ecosystems.

In the Jiaodong region, linear regression (LR) demonstrated limited predictive capability (R² = 0.57, RMSE = 0.02), exhibiting systematic residuals, such as underestimating high-SOC conditions and overestimating low-SOC microsites (**Figure 7a**). In contrast, both support vector machine (SVM; R² = 0.99, RMSE = 0.017) and XGBoost (R² = 0.99, RMSE = 0.02) models exhibited superior overall accuracy, though they displayed minor deviations at extreme SOC values (**Figures 7c, d**), due to reduced robustness in capturing complex interactions such as dual-threshold NO_3_^-^-N and Fe co-limitation. In Southwest Shandong, the predominantly linear relationship between total nitrogen (TN) and SOC allowed the linear regression (LR) model to achieve a relatively high coefficient of determination (R² = 0.97; **Figure 7e**). However, the model exhibited a substantially higher RMSE (0.55) compared to the random forest (RF) model (0.01; **Figure 7f**), indicating its limited capacity to capture the nonlinear interactions between TN and cation exchange capacity (CEC) that drive SOC stabilization. The support vector machine (SVM) model demonstrated reduced predictive accuracy at elevated SOC levels (RMSE = 0.35; **Figure 7g**), particularly under conditions where TN exceeded 4.0 g·kg^-^¹ and CEC was below 12 cmol·kg^-^¹. By maintaining near-optimal precision (R² = 0.99, RMSE = 0.03; **Figure 7h**), the XGBoost model effectively captured the underlying complexity of biogeochemical interactions, indicating its robustness in modeling multidimensional SOC dynamics.

## 4 Discussion

### 4.1 Spatial variability of SOC in the Jiaodong Peninsula and Southwestern Shandong Province

The Jiaodong Peninsula, under the effect of maritime monsoons, and Southwestern Shandong, characterized by a typical continental climate, exhibit fundamental differences in the spatial variability of SOC. In the Jiaodong Peninsula, SOC exhibits relatively low spatial variability, as indicated by strong spatial autocorrelation (*p* < 0.001; nugget effect = 39.20%) and a short range (~42.6 km; **Supplementary Table S1**), suggesting that its heterogeneity is predominantly controlled by local-scale factors such as soil type and land-use practices. Probability analysis further reveals that SOC concentrations across the Jiaodong Peninsula are substantially lower than those in Southwestern Shandong, particularly in the eastern coastal areas where high Fe content and low NO_3_^-^–N concentrations (<10 mg·kg^-^¹) coincide with a 20–60% probability of a 10–20 g·kg^-^¹ SOC deficit (**Figures 2a, c**; **Supplementary Figure S1**). These observations indicate that well-aerated, oxidizing environments and rapid organic matter mineralization, coupled with the destabilization of organo-mineral complexes induced by high Fe contents, may contribute to the reduced SOC levels in this region (Keiluweit et al., 2015; Chen et al., 2020).

By contrast, SOC in Southwestern Shandong exhibits more distinct spatial heterogeneity. The semivariogram exhibits a longer range (68.00 km) and a very low nugget effect (1.12%), indicating that large-scale structural factors-such as gradients in total nitrogen (TN) and cation exchange capacity (CEC), play a dominant role in shaping SOC distribution. Specifically, high SOC values (>25 g·kg^-^¹) are concentrated in the southeastern part of the region, closely associated with elevated TN (>4 g·kg^-^¹) and CEC levels (>12 cmol·kg^-^¹) (**Figure 3a** and **Supplementary Figure S2**). This spatial co-occurrence likely reflects the joint stabilization of organo-mineral complexes via nitrogen enrichment and cation bridging mechanisms, leading to the formation of SOC-enriched domains (Rowley et al., 2018a). Probability analysis shows that across most of the region, the probability of SOC concentrations exceeding those in the Jiaodong Peninsula is as high as 80-100% (**Figure 3**), with substantial areas exhibiting differences of 10–20 g·kg^-^¹, particularly in the southeastern regions. In contrast, the spatial heterogeneity observed in the northwestern low-SOC regions may be closely linked to enhanced physical erosion associated with steeper slopes (**Figure 3a** and **Supplementary Figure S2e**), which preferentially transports fine organic and mineral particles, thereby reducing local SOC stability (Van Oost et al., 2007).

Sequential Gaussian simulation further quantifies uncertainty in SOC spatial prediction. The mean and standard deviation maps reveal an evident west-high-east-low gradient in the Jiaodong Peninsula, with the spatial distribution of SOC standard deviation corresponding to the high-SOC western regions (**Figures 4a–c**). In contrast, the eastern coastal region, characterized by lower SOC levels, exhibits significantly elevated simulation uncertainty compared to the central-western uplands, implying that fertilization and land management practices highlight the contribution of site-specific fertilization regimes and management heterogeneity to microscale SOC variability. In Southwestern Shandong, SOC prediction uncertainty exhibits a discontinuous threshold response at 3.25 g·kg^-^¹ TN, with standard deviations exceeding 5 g·kg^-^¹ in low-TN areas (<3 g·kg^-^¹) but declining to values under 2.5 g·kg^-^¹ in high-TN areas (>4 g·kg^-^¹), indicating that uncertainty is largely driven by spatial variation in soil nitrogen availability. Moreover, the coefficient of variation (CV; STD/Mean) in the northwestern part of Southwestern Shandong (CV; 0.35-0.42) is significantly higher than in the southeast (CV; 0.12-0.18) (**Figures 4d,e**), likely due to its location within a nitrate-rich input region (>27 mg·kg^-^¹, **Supplementary Figure S2d**), where complex feedbacks involving denitrification and the breakdown of mineral-associated carbon substantially increase the dynamic variability and spatial heterogeneity of SOC (Giannetta et al., 2019; Chen et al., 2020; Song et al., 2023).

### 4.2 Coupled regulation of nitrate and extractable iron on SOC stability under Maritime Monsoon Conditions in the Jiaodong Peninsula, Shandong Province

Nitrate nitrogen (NO_3_^-^-N) and extractable iron are established as the dominant controls for the stability of soil organic carbon (SOC) in the Jiaodong Peninsula (**Figures 5a,b**). Notably, elevated NO_3_^-^-N concentrations enhance SOC stability in this region (**Figure 5c**), while extractable iron exhibits a distinctly nonlinear regulatory pattern, enhancing SOC stability at low concentrations but reducing it at higher levels (**Figures 5b, d**; SHAP value = -0.82 ± 0.05). The interaction between NO_3_^-^-N and extractable iron further increases the complexity, leading to a concentration-dependent dual-threshold response to changes in NO_3_^-^-N concentration with respect to SOC stability.

These findings highlight a critical limitation of conventional single-factor fertilization strategies, as soils situated within the identified dual-threshold domain exhibit pronounced sensitivity and reduced stability in SOC responses. Under such conditions, even moderate nitrogen additions can induce transitions in stabilization dynamics, shifting SOC from net losses to net gains. This nonlinearity indicates the potential risks of nutrient management decisions that rely on soil nitrogen status without consideration of the iron context, which may lead to reductions in SOC persistence. To mitigate this risk, a shift is required from single-nutrient threshold management toward integrated, multi-factor nutrient regulation. From an applied perspective, this requires the incorporation of extractable Fe as a diagnostic criterion in nitrogen management and the adoption of more conservative application strategies-such as split fertilization-to maintain NO_3_^-^-N concentrations within ranges that promote SOC stabilization. Therefore, the integrated framework provides a robust scientific basis for enhancing soil carbon sequestration in regional croplands while advancing precision agriculture approaches that explicitly incorporate pedoclimatic and edaphic heterogeneity.

It is important to emphasize that while these model-derived interactions provide substantial evidence for synergistic regulation, they fundamentally represent statistical dependencies captured by the algorithm. Therefore, they should be interpreted as strong hypotheses for underlying biogeochemical mechanisms that require specific experimental validation in future research. The underlying mechanism thus suggested likely involves a shift in soil biogeochemical processes induced by varying NO_3_^-^-N concentrations. At levels below 10 mg·kg^-^¹, NO_3_^-^-N serves as an electron acceptor in denitrification, leading to reducing conditions that thermodynamically favor the reduction of Fe (III) to Fe (II), thereby disrupting Fe-C complexes and releasing formerly protected organic carbon, ultimately decreasing SOC stability (Ding et al., 2015; Song et al., 2023). Conversely, when NO_3_^-^-N exceeds this threshold, microbial metabolism likely shifts from anaerobic to aerobic respiration. For example, elevated nitrate concentrations may enhance microbial diversity and stimulate extracellular enzyme activities (e.g., oxidases and cellulases), accelerating organic matter decomposition and transformation, while simultaneously promoting microbial necromass accumulation and facilitating the “restabilization” of SOC (Liang et al., 2017). In addition, changes in nitrogen stoichiometry (e.g., decreased C/N ratio) and NO_3_^-^-N-induced mineral-organic interactions-such as the increased adsorption of microbial metabolites to iron oxides-may improve the physical protection of SOC within microaggregates (Lehmann et al., 2020). Therefore, the positive role of NO_3_^-^-N on SOC becomes evident only beyond a certain concentration threshold, indicating both a “concentration dependence” and an “ecological context dependence” in the nitrate-SOC regulatory mechanism. These findings also suggest that nitrate availability at or above the threshold level (≥10 mg·kg^-^¹) constitutes an essential condition for activating the positive regulatory role of iron in SOC dynamics and sustaining long-term SOC stabilization.

### 4.3 Total nitrogen-driven SOC accumulation threshold mechanism and multifactor joint regulation under continental climate in Southwestern Shandong Province

Under the typical continental climatic conditions in Southwestern Shandong, total nitrogen (TN) is identified as the dominant factor governing soil organic carbon (SOC) stability (**Figures 6a,b**). Both univariate regression analysis and the random forest model reveal a significant positive relationship between TN and SOC (adjusted *R*² = 0.28, β = 2.89, p < 0.001; **Figure 6c**). Partial dependence analysis shows that beyond the critical TN threshold (3.25 ± 0.11 g·kg^-^¹), predicted SOC undergoes a discontinuous regime shift with a net increase of 2.0 g·kg^-^¹ (ΔSOC/ΔTN = 1.54, **Figure 6d**). This threshold response likely indicates that TN accumulation enhances plant productivity and litter inputs while increasing nitrogen content, thereby reducing the soil C/N ratio, improving microbial carbon use efficiency, promoting microbial necromass carbon accumulation and macroaggregate carbon sequestration, and ultimately enhancing SOC stabilization (Hu et al., 2022; Zhao et al., 2022; Zhou et al., 2023). Spatially, the median TN value is 3.67 g·kg^-^¹, which lies to the right of the threshold turning point, indicating that nearly half of the soils in the region remain within the nitrogen-facilitated SOC accumulation domain.

In contrast, nitrate nitrogen (NO_3_^-^-N) exhibits a typical negative threshold effect. When its concentration exceeds 27 mg·kg^-^¹, predicted SOC values decline by 3.1% (**Figure 6e**). SHAP analysis further demonstrates that in high NO_3_^-^-N regions (>40 mg·kg^-^¹), the marginal contribution of NO_3_^-^-N to SOC is negative (SHAP = -1.2; **Figures 6b, e**), indicating a reduction in SOC stabilization. This decline results from the enhanced denitrification under elevated nitrate availability, which reduces soil redox potential and facilitates the reduction of Fe(III) to Fe(II), thereby destabilizing Fe-C complexes and releasing protected organic carbon (Chen et al., 2020). Moreover, under high NO_3_^-^-N or acidic conditions, Ca-organic matter (Ca-C) complexes may also be destabilized (Rowley et al., 2018a; Shabtai et al., 2023; Rowley et al., 2025), leading to a decrease of mineral protection and a greater SOC bioavailability. Elevated NO_3_^-^-N enhances SOC stability in the Jiaodong Peninsula, whereas it reduces SOC stability in Southwestern Shandong. This contrast can be explained by distinct soil background conditions in the two regions. The Jiaodong Peninsula, under a maritime monsoon climate, is characterized by soils with high oxygen availability and dynamic redox conditions. Therefore, NO_3_^-^-N inputs promote a shift from anaerobic to aerobic microbial metabolism, thereby stimulating extracellular enzyme activity and microbial necromass formation, which together contribute to a biologically driven pathway of SOC stabilization dominated by microbial carbon assimilation and aggregate protection (Giannetta et al., 2019; Ali et al., 2021; Hou et al., 2024). In contrast, the low precipitation and clay-rich soils in Southwestern Shandong limit oxygen diffusion in surface layers, resulting in widespread localized anaerobic microsites. Under these conditions, high NO_3_^-^-N levels induce denitrification-reduction processes that promote SOC destabilization through mineral-associated carbon (MAOC) release, which is indicative of a denitrification-mineral destabilization mechanism (Song et al., 2023). Thus, the effect of NO_3_^-^-N on SOC is context-dependent, reflecting strong regional specificity driven by distinct soil-climate conditions.

Apart from nitrogen, other nutrient factors also exhibit significant nonlinear and interactive effects on the spatial variability of SOC in Southwestern Shandong. Total phosphorus (TP), for instance, displays a dual negative threshold at 0.71 and 0.81 g·kg^-^¹, with SOC reductions of 2.0-4.0 g·kg^-^¹ (**Figure 6f**). The regional median NO_3_^-^-N level (14.94 mg·kg^-^¹) approaches the SOC response threshold (27 mg·kg^-^¹), while the TP median coincides with the first critical threshold (0.71 g·kg^-^¹), constituting co-occurrence domains - “high NO_3_^-^-high TP”, where NO_3_^-^-driven Fe reduction and phosphate ligand-exchange destabilize Fe-C associations (**Supplementary Figure S2b**, S2d). This risk region may enhance Fe(III) reduction through NO_3_^-^-induced denitrification, while phosphate competes for adsorption sites, further disrupting Fe-C complex stability and promoting MAOC destabilization and SOC loss (Antelo et al., 2007; Fu et al., 2013; Song et al., 2023). SHAP interaction analysis in **Figure 6h** further reveals a nonlinear enhancement between TN and cation exchange capacity (CEC). When TN levels are lower than 4.0 g·kg^-^¹, the contributions of TN and CEC to SOC are negative, suggesting that limited nitrogen supply and low CEC jointly restrict SOC formation. However, once TN exceeds this threshold, increasing CEC significantly enhances its positive effect on SOC. This shift is consistent with the dual-phase SOC response observed at CEC levels of 9.8 and 11.9 cmol·kg^-^¹, with a maximum SOC of 3.2 g·kg^-^¹ (**Figure 6g**). Such transitions may be driven by Ca²^+^/Mg²^+^-mediated cation bridging mechanisms activated within optimal CEC ranges, facilitating carboxyl–clay complexation and thereby enhancing the stability of organo-mineral associations at the aggregate scale (Rowley et al., 2018a). Simultaneously, sufficient TN supply provides essential precursors for microbial necromass accumulation and mineral-associable carbon inputs, thus supporting the microbial pathway of SOC formation (Liao et al., 2020; Zhou et al., 2023; Lv et al., 2024).

### 4.4 Advantages of machine learning in SOC prediction and mechanistic interpretation

This study demonstrates predictive capacity of four machine learning models-linear regression (LR), random forest (RF), XGBoost (XGB) and support vector machine (SVM)-in capturing nonlinear dynamics of SOC (**Figure 7**). The random forest consistently exhibited optimal performance across both study regions (adjusted R² = 0.99; RMSE = 0.005 in Jiaodong and 0.01 in Southwest Shandong; **Figures 7b, f**), attributed to its ability to decouple complex multivariate interactions (Breiman, 2001). These findings demonstrate the capacity of machine learning to capture spatial heterogeneity in ecological variables through non-parametric approaches. Importantly, the advantages of machine learning extend beyond predictive precision. In addition to predictive performance, the interpretive components of machine learning algorithms facilitate a process-based understanding of the biogeochemical mechanisms that regulate SOC formation. The machine learning approaches enable dual validation that optimizes both predictive accuracy and mechanistic interpretation, thereby establishing a data-driven framework for ecosystem process research. For example, variable importance ranking and partial dependence analysis in Random Forest jointly revealed a dual-threshold interaction between NO_3_^-^-N and extractable Fe in Jiaodong, as determined by SHAP values (SHAP = 0.32; Zeraatpisheh et al., 2019). In Southwest Shandong, SOC exhibited a substantial threshold shift (ΔSOC = 12.3 g·kg^-^¹) at a TN concentration of 4.0 g·kg^-^¹ and captured a joint effect of TN and cation exchange capacity (CEC). This multidimensional mechanistic analysis not only demonstrates the methodological significance of interpretable machine learning but also provides a critical perspective on the portability of predictive models. The observation that a complex random forest model was indispensable for capturing interactive dynamics in the Jiaodong region, whereas simpler linear models yielded partial explanatory power under the TN-dominated regime of Southwest Shandong (**Figure 7**), indicates that the suitability of model structures is inherently constrained by their pedoclimatic context. These findings highlight the risks associated with applying a uniform modeling framework across heterogeneous environments and emphasize the necessity of regionally calibrated approaches to achieve robust predictions of ecosystem processes (Chen and Guestrin, 2016).

Overall, it is important to acknowledge that our study was intentionally restricted to the topsoil (0–20 cm), whereas a substantial proportion of the total SOC stock is retained in deeper horizons, where stabilization mechanisms may differ fundamentally from those identified here (Jobbágy and Jackson, 2000). In subsoils, the dominant controls on SOC are likely to shift from biologically mediated, nutrient-dependent dynamics (e.g., NO_3_^-^-N, total N) that prevail in the surface layer towards processes primarily governed by physicochemical protection. Accordingly, the threshold responses identified in this study may give way to alternative critical thresholds associated with clay content, the saturation of reactive mineral surfaces (e.g., Fe/Al oxides), or cation-bridging capacity. Applying the integrative analytical framework developed herein-combining geostatistics with interpretable machine learning-to entire soil profiles therefore constitutes a crucial next step, one that could provide a more comprehensive, spatially explicit understanding of SOC persistence and its controlling mechanisms across contrasting pedoclimatic regimes.

## 5 Conclusion

To conclude, the combination of machine learning models with geostatistical approaches enables the identification of spatial patterns and threshold mechanisms, with maritime regions exhibiting dual Fe-NO_3_^-^-N interactions and continental regions showing nitrogen-dominated threshold responses that regulate SOC dynamics across contrasting pedoclimatic gradients. The spatial structure of SOC variability exhibits distinct patterns between pedoclimatic regions, with local-scale heterogeneity characterized by strong spatial autocorrelation and shorter range in Jiaodong Peninsula (maritime monsoon climate), while macro-scale gradients dominated by total nitrogen and cation exchange capacity are observed in Southwest Shandong (continental climate) with longer range and lower nugget effect. In maritime regions, a dual-threshold response pattern was identified wherein the relationship between iron and SOC stability exhibited opposing patterns regulated by nitrate availability, indicating that biogeochemical interactions rather than individual factors determine SOC dynamics, whereas in continental regions, nitrogen-mediated threshold responses predominate, with nitrate demonstrating contrasting effects relative to maritime conditions, thus highlighting the pedoclimatic specificity of nutrient regulation under varying soil redox regimes. The successful application of SHAP analysis in quantifying nonlinear interactions and elucidating the joint effects of nutrients and soil properties demonstrates the value of interpretable machine learning in advancing mechanistic understanding of complex soil-environment relationships beyond traditional linear approaches, with important implications for region-specific SOC management. In the Jiaodong Peninsula, maintaining an appropriate nitrate supply is critical for reducing the susceptibility of SOC to losses under variable redox conditions, whereas in Southwest Shandong, enhancing cation exchange capacity and ensuring sufficient TN levels are essential for strengthening SOC stability. The empirically derived mechanisms critically challenge the structural simplifications widely applied in Earth system models and demonstrate the requirement to incorporate explicit biogeochemical processes in order to strengthen their capacity to simulate future climate-carbon feedbacks. The results further provide a basis for developing region-specific soil carbon management frameworks that integrate threshold responses, biogeochemical interactions, and pedoclimatic constraints, thereby replacing single-factor approaches with a process-oriented perspective.

## Data Availability

The original contributions presented in the study are included in the article/**Supplementary Material**. Further inquiries can be directed to the corresponding authors.
