# Supplementary material for: Nonlinear threshold responses and spatial heterogeneity of soil organic carbon under contrasting pedoclimatic regimes
Source: Front Plant Sci. 2025 Dec 9;16:1703663. doi: 10.3389/fpls.2025.1703663 (PMC12722956; doi:10.3389/fpls.2025.1703663)
Supplement: Supplementary file 1 [file Table1.docx]

**Table S1** Variogram model parameters for soil organic carbon (SOC) and associated environmental factors in the Jiaodong Peninsula and Southwest Shandong.

| Region | Indicators | | Variogram  Model | Nugget(C_O_) | Sill(C_O_+C) | C_O_/(C_O_+C)/% | Range/km | R^2^ | RSS |
| --- | --- | --- | --- | --- | --- | --- | --- | --- | --- |
| Jiaodong Region | SOC/(g•kg^-1^) | | Exponential | 1.67 | 4.26 | 39.20 | 42.60 | 0.22 | 10.00 |
|  | Probability of Acidification | ΔSOC/(g•kg^-1^)<-20 | Gaussian | 8 | 206.80 | 3.87 | 111.54 | 0.99 | 220 |
|  |  | -20≤ΔSOC/(g•kg^-1^)<-10 | Exponential | 0.01 | 18.58 | 0.05 | 189.90 | 0.99 | 1.47 |
|  |  | -10≤ΔSOC/(g•kg^-1^)<-6 | Exponential | 0.01 | 14.33 | 0.07 | 109.80 | 0.99 | 1.56 |
|  |  | -6≤ΔSOC/(g•kg^-1^)<0 | Spherical | 0.1 | 33.56 | 0.30 | 123.50 | 0.99 | 7.21 |
|  |  | 0≤ΔSOC/(g•kg^-1^) | Spherical | 0.1 | 108.30 | 0.09 | 146.90 | 0.99 | 57.3 |
|  | mean value of SOC | | Gaussian | 1.16 | 18.21 | 6.37 | 115.18 | 0.99 | 3.13 |
|  | SOC standard deviation | | Exponential | 0.001 | 0.01 | 10 | 83.70 | 0.99 | 6.27E-07 |
|  | NO₃⁻-N/(mg•kg^-1^) | | Gaussian | 1.00 | 411.80 | 0.245 | 19.05 | 0.36 | 234545 |
|  | TE-Fe/(mg•kg^-1^) | | Gaussian | 21.42 | 42.85 | 49.99 | 96.65 | 0.42 | 1150 |
|  | Predict SOC  /(g•kg^-1^) | | Gaussian | 0.001 | 1.07 | 0.09 | 19.40 | 0.40 | 1.18 |
| The southwesten Shandong. | SOC/(g•kg^-1^) | | Spherical | 0.60 | 53.74 | 1.12 | 68 | 0.89 | 260 |
|  | Probability of Acidification | ΔSOC/(g•kg^-1^)<-6 | Exponential | 0.1 | 201.1 | 0.05 | 269.40 | 0.97 | 703 |
|  |  | -6≤ΔSOC/(g•kg^-1^)<0 | Spherical | 1 | 476.40 | 0.21 | 143.90 | 0.99 | 369 |
|  |  | 0≤ΔSOC/(g•kg^-1^)<6 | Spherical | 1 | 438.60 | 0.23 | 75.00 | 0.99 | 3692 |
|  |  | 6≤ΔSOC/(g•kg^-1^)<10 | Spherical | 0.1 | 215.5 | 0.05 | 43.20 | 0.94 | 3327 |
|  |  | 10≤ΔSOC/(g•kg^-1^)<20 | Spherical | 3 | 885.7 | 0.34 | 93.50 | 0.99 | 4933 |
|  |  | 20≤ΔSOC/(g•kg^-1^) | Gaussian | 31 | 615.70 | 5.03 | 48.32 | 0.99 | 1564 |
|  | mean value of SOC | | Gaussian | 2.8 | 94.06 | 2.98 | 95.61 | 0.99 | 24.50 |
|  | SOC standard deviation | | Spherical | 0.001 | 2.66 | 0.04 | 141.90 | 0.99 | 0.02 |
|  | TN/(g•kg^-1^) | | Spherical | 0.001 | 2.52 | 0.04 | 64.60 | 0.94 | 0.32 |
|  | TP/(g•kg^-1^) | | Spherical | 0.0001 | 0.22 | 0.05 | 73.90 | 0.90 | 5.15E-03 |
|  | CEC | | Gaussian | 0.01 | 22.58 | 0.04 | 27.71 | 0.62 | 232 |
|  | [NO_3_^-^-N](https://zhidao.baidu.com/question/45872866.html)/(mg •kg^-1^) | | Spherical | 104 | 967.00 | 10.75 | 64.6 | 0.92 | 44247 |
|  | Predict SOC  /(g•kg^-1^) | | Exponential | 2.44 | 25.2 | 9.68 | 0.60 | 0 | 430 |
