# Supplementary material for: Nonlinear threshold responses and spatial heterogeneity of soil organic carbon under contrasting pedoclimatic regimes
Source: Front Plant Sci. 2025 Dec 9;16:1703663. doi: 10.3389/fpls.2025.1703663 (PMC12722956; doi:10.3389/fpls.2025.1703663)
Supplement: Supplementary file 2 [file DataSheet1.pdf]

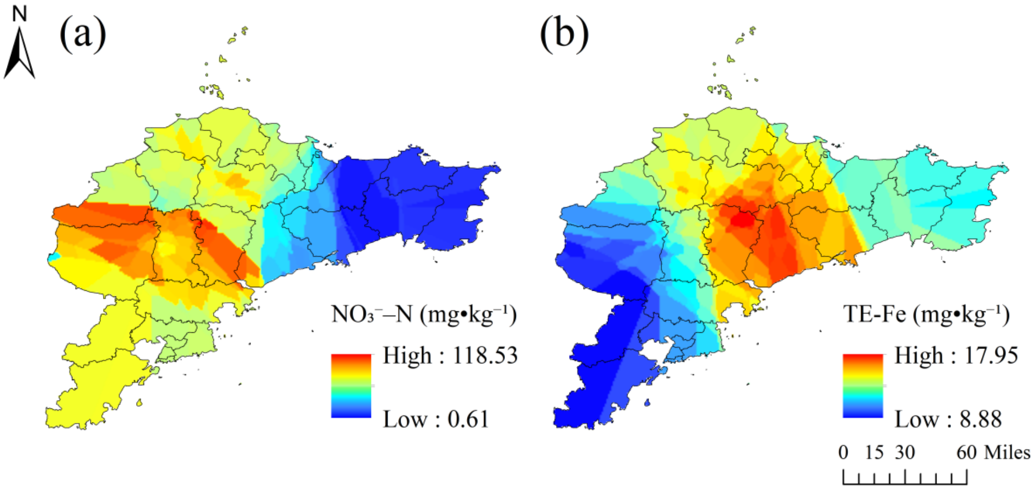

Fig. S1. Dominant factors controlling soil organic carbon in the Jiaodong Peninsula: (a) nitrate nitrogen (NO_3^--N) and (b) DTPA-extractable iron (TE-Fe).

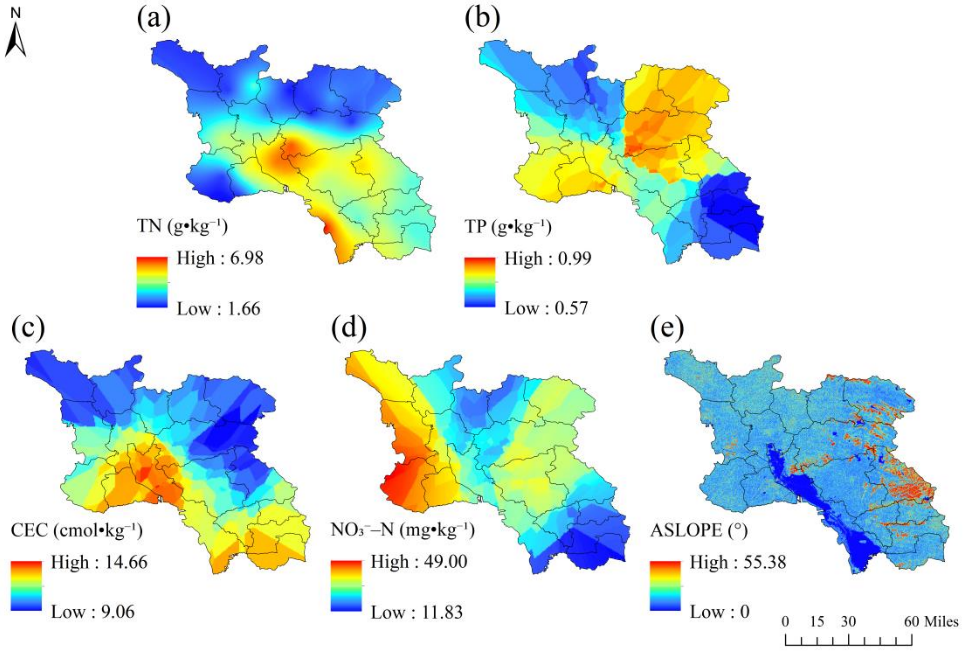

Fig. S2. Dominant factors controlling soil organic carbon in Southwest Shandong: (a) total nitrogen (TN), (b) total phosphorus (TP), (c) cation exchange capacity (CEC), (d) nitrate nitrogen (NO_3^--N), and (e) ASLOPE (°)
